# A 25 micron-thin microscope for imaging upconverting nanoparticles with NIR-I and NIR-II illumination

**DOI:** 10.7150/thno.37672

**Published:** 2019-10-18

**Authors:** Hossein Najafiaghdam, Efthymios Papageorgiou, Nicole A. Torquato, Bining Tian, Bruce E. Cohen, Mekhail Anwar

**Affiliations:** 1 Department of Electrical Engineering and Computer Sciences, University of California Berkeley, Berkeley CA; 2 The Molecular Foundry, Lawrence Berkeley National Laboratory, Berkeley, CA; 3 Department of Radiation Oncology, University of California San Francisco, San Francisco, CA

**Keywords:** intraoperative imaging, upconverting nanoparticle, time-resolved imaging, NIR excitation

## Abstract

**Rationale:** Intraoperative visualization in small surgical cavities and hard-to-access areas are essential requirements for modern, minimally invasive surgeries and demand significant miniaturization. However, current optical imagers require multiple hard-to-miniaturize components including lenses, filters and optical fibers. These components restrict both the form-factor and maneuverability of these imagers, and imagers largely remain stand-alone devices with centimeter-scale dimensions.

**Methods:** We have engineered *INSITE* (Immunotargeted Nanoparticle Single-Chip Imaging Technology), which integrates the unique optical properties of lanthanide-based alloyed upconverting nanoparticles (aUCNPs) with the time-resolved imaging of a 25-micron thin CMOS-based (complementary metal oxide semiconductor) imager. We have synthesized core/shell aUCNPs of different compositions and imaged their visible emission with INSITE under either NIR-I and NIR-II photoexcitation. We characterized aUCNP imaging with INSITE across both varying aUCNP composition and 980 nm and 1550 nm excitation wavelengths. To demonstrate clinical experimental validity, we also conducted an intratumoral injection into LNCaP prostate tumors in a male nude mouse that was subsequently excised and imaged with INSITE.

**Results:** Under the low illumination fluences compatible with live animal imaging, we measure aUCNP radiative lifetimes of 600 μs - 1.3 ms, which provides strong signal for time-resolved INSITE imaging. Core/shell NaEr_0.6_Yb_0.4_F_4_ aUCNPs show the highest INSITE signal when illuminated at either 980 nm or 1550 nm, with signal from NIR-I excitation about an order of magnitude brighter than from NIR-II excitation. The 55 μm spatial resolution achievable with this approach is demonstrated through imaging of aUCNPs in PDMS (polydimethylsiloxane) micro-wells, showing resolution of micrometer-scale targets with single-pixel precision. INSITE imaging of intratumoral NaEr_0.8_Yb_0.2_F_4_ aUCNPs shows a signal-to-background ratio of 9, limited only by photodiode dark current and electronic noise.

**Conclusion:** This work demonstrates INSITE imaging of aUCNPs in tumors, achieving an imaging platform that is thinned to just a 25 μm-thin, planar form-factor, with both NIR-I and NIR-II excitation. Based on a highly paralleled array structure INSITE is scalable, enabling direct coupling with a wide array of surgical and robotic tools for seamless integration with tissue actuation, resection or ablation.

## Introduction

Highly sensitive imaging in hard-to-access areas remains a persistent challenge for optical surgical navigation. This is increasingly prevalent in the modern era of minimally invasive cancer surgeries where visualizing microscopic disease over the entirety of a small, complex tumor bed is hindered by the form-factor of intraoperative optical imagers, and consequently microscopic residual disease (MRD) is common 1. MRD significantly increases cancer recurrence 2, 3, necessitating substantial additional treatment, resulting in additional toxicity and cost. Despite these efforts, clinical outcomes of additional therapy often remain inferior to complete resection upfront 4. MRD remains common despite the advent of new targeted molecular imaging agents 5 and intraoperative optical imagers 6-8.

Intraoperative imagers have made significant progress for precision guided surgeries 7, 8. Leveraging the growing number of specific optical probes to label tumor cells *in vivo*, stand-alone widefield fluorescence microscopes are increasingly common for surgical guidance 6, and advances in engineering have decreased sizes to centimeter scales 9-16. While intraoperative imagers have had significant impacts on surgical outcomes, current designs are limited in both miniaturization and adaptability. In particular, current fluorescent probes that target proteins or cells necessitate the use of rigid, bulky optical lenses to distinguish probe emission from excitation light 17. This need for rigid optical components has been a barrier to miniaturization. While advances in microfabrication have achieved small lenses 18-20, fundamental physical limits and fabrication challenges hinder further miniaturization, and ultra-small lenses face challenges in producing high resolution images. Furthermore, the requirement for precise positioning of the tissue sample in the focal plane and imaging over a broad area hinder utilization of these micro-scale lenses intraoperatively. Optical lenses are not easily planarized - precluding an ultra-thin form-factor necessary for broad device integration. Fiber-optic based imagers address this problem by guiding light out of the tumor bed to a large standalone imager, but fundamental tradeoffs between imaging area and flexibility hinder achieving both high maneuverability and rapid imaging of the entire tumor cavity.

Chip-based imagers eliminate the need for optics in favor of contact imaging 21, 22 placing the imager in direct tissue contact to capture light before it diverges, microfabricated patterns to focus light 23, or computational approaches 24-26. While these imagers are significantly more compact without the use of lenses, the high-performance optical filters required to image fluorophores with small Stokes shifts demands precision optical alignment 27, 28, substantially hindering further miniaturization for intraoperative imaging.

Chip-based tissue imagers built around optical probes that enable time-resolved imaging 29, 30 would obviate the need for both filters and lenses, and allow for exceptionally small and flexible microscopes. To date this approach has been limited for tissue imaging by both the nanosecond radiative decay times of organic optical labels, and the similarity of decay times with tissue autofluorescence, masking small signals with background. Imager sensitivity is fundamentally limited by scattering 31 and cellular autofluorescence 32, 33 , which is strongest with UV and visible excitation and cannot be removed with filtering 34, 35. Optical labels with longer lifetimes and photoexcitation at longer wavelengths in the NIR-I (700 - 1000 nm) or NIR-II (typically, 1000 - 1700 nm) 36 might circumvent these problems.

As long-lifetime optical probes, lanthanide-based upconverting nanoparticles (UCNPs) are able to sum the energies of multiple NIR photons and emit at higher energies in the NIR or visible spectrum. UCNP luminescence efficiencies are up to 10 orders of magnitude higher than those of the best 2-photon fluorophores 37, 38 and show no overlap with cellular autofluorescence, no measurable photobleaching, even under prolonged single-particle excitation 39-42. UCNPs make use of energy transfer upconversion between neighboring lanthanide ions, in which sensitizer ions sequentially transfer absorbed energy to luminescent emitter ions. Numerous studies have shown that the sensitizer/emitter pair of Yb^3+^/Er^3+^ doped into host matrix NaYF_4_ nanocrystals is most efficient and emits both green and red light with either continuous wave NIR-I (980 nm) or NIR-II (1550 nm) lasers. Recent work has shown that alloyed upconverting nanoparticles (aUCNPs), in which host matrix metals are replaced entirely with lanthanides, are significantly brighter than their doped counterparts, particularly at low laser fluences compatible with living systems 37. A key advantage of UCNPs is that they can be excited with low fluences of either NIR-I or NIR-II light, minimizing interactions with both cells and semiconductors, while emitting at the visible wavelengths appropriate for most common imaging detectors. Human maximum permitted exposures 43 are ~200 W/cm^2^ for 5-ms pulses of either 980 nm or 1550 nm light (Table S1), suggesting NIR-excitable probes can be imaged during surgery with minimal phototoxicity.

In this study, we have integrated both custom integrated circuit imager design with nanoparticle engineering, and introduced INSITE, a 25 micron-thin microscopic imaging platform for imaging aUCNPs. We achieve this level of miniaturization by eliminating conventional fluorophores and their requisite optics in favor of a scalable, planar chip-based microscope, custom designed and fabricated in a 0.18-micron complementary metal oxide semiconductor (CMOS) process, in synergistic combination with long-lifetime NIR I and II-excited upconverting nanoparticles. To determine the optimal UCNPs composition to use with INSITE, we first investigate three different types of lanthanide-based aUCNPs and we subsequently present experimental results obtained using INSITE from an intratumorally-injected mouse prostate tumor specimen.

## Materials and Methods

### Imager design and fabrication

An ultra-thin imager array was designed as an application-specific integrated circuit (ASIC) imaging array consisting of 2,880 pixels 44. The resulting INSITE chips were custom fabricated in a 0.18 μm CMOS process, and thinned down to 25 μm. Each pixel contains a silicon photodiode (44 μm 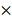 44 μm), followed by a 4-transistor front-end amplifier which enables a current integration over a custom-made MOM (metal-oxide-metal) capacitor. A subsequent sample-hold block controls pixel timing.

### Eliminating lenses and angle-selective gratings

To obtain spatial resolution with minimum form-factor, we used on-chip angle-selective gratings using designs similar to those previously reported 22, 44, 45. Angle selective gratings are an array of microfabricated collimators patterned directly over each photodiode, effectively coupling each photodiode to the tissue directly opposite it, blocking obliquely incident background light.

### Nanocrystal synthesis

**Growth of aUCNP cores**: aUCNPs were grown as previously described 37, 46 For, 8-nm NaEr_0.6_Yb_0.4_F_4_ cores: YbCl_3_ hexahydrate (64 mg, 0.16 mmol) and ErCl_3_ (66 mg, 0.24 mm) were stirred in oleic acid (OA, 3.25 g, 10.4 mmol) and 4 mL of ODE and heated under vacuum for 1 h at 110 °C. The reaction was cooled to room temperature under N_2_, followed by the addition of sodium oleate (381 mg, 1.25 mmol), NH_4_F (74 mg, 2.0 mmol) and 3 mL of ODE. The reaction was then held under vacuum for 20 min, followed by 3 cycles of refilling with N_2_ and purging. The reaction temperature was then increased to 315 °C. After 45 min, nanocrystal growth was halted by removing the heating mantle and cooling the flask to 75 °C with a stream of air, followed by the addition of 20 mL of EtOH and 20 mL of acetone. The UCNPs were precipitated twice with EtOH and redispersed in 10 mL of hexane with 0.2% (*v*/*v*) OA.

**Growth of core/shell aUCNPs:** Epitaxial 4-nm NaY_0.8_Gd_0.2_F_4_ shells were overgrown using adaption of a layer-by-layer method 46. A 0.10 M solution of 80:20 Y/Gd oleate (Y/Gd-OA) was prepared by heating YCl_3_ (78 mg, 0.40 mmol) and GdCl_3_ (26 mg, 0.10 mmol) to 110 °C in OA (2 mL) and ODE (3 mL) and stirred for 15 min under vacuum. The flask was filled with N_2_ and heated to 160 °C for 30 min, followed by another 15 min at 110 °C under vacuum. In a separate flask, a 0.40 M NaTFA-OA precursor solution was prepared by dissolving sodium trifluoroacetate (163 mg, 1.20 mmol) in oleic acid (3 mL) and applying vacuum at room temperature for 20 min.

Purified aUCNP cores (27 μmol) in hexane were added to 4 mL of OA and 6 mL of ODE. The mixture was held under vacuum for 30 min at 70 °C to remove the hexane. The shell growth was performed under N_2_ at 280 °C, with alternating injections of Y/Gd-OA and NaTFA-OA precursor performed at 15 min intervals. After the last injection, the reaction was maintained at 280 ° C for an additional 30 min to allow for complete shell growth, followed by rapid cooling. Nanoparticles were purified and stored as described for the aUCNP cores.

**UCNP polymer encapsulation**: Hydrophobic core/shell aUCNPs were dispersed in hexane with 0.2% (*v*/*v*) oleic acid to 5 μM. For aqueous dispersions 37, 46, 6 mg of poly(maleic anhydride-*alt*-1-octadecene) copolymer (MW 20-25k, Aldrich) was dissolved to 17 μM in 0.5 mL of acetone and 15 mL of CHCl_3_. aUCNPs (0.5 nmol) in 100 μL of hexane were added with stirring, and the solvents were removed under a gentle stream of N_2_ overnight. The aUCNP/polymer residue was resuspended in a solution of MeO-PEG_8_-NH_2_ (ThermoFischer, 10 μmol) in 10 mL of 100 mM NaHCO_3_ buffer, pH 8.2, with 1% (*v*/*v*) DMSO. This suspension was sonicated for 60 min, heated in an 80 °C water bath for 60 min, slowly cooled to room temperature, and then sonicated for 30 min. Excess polymer was removed by spin dialysis (Amicon, 100 kDa MWCO), washing with 4×15 mL of 100 mM HEPES, pH 7.4. Retained aUCNPs were concentrated to 680 μL and filtered through a 0.2-μm filter into a sterile glass vial.

### Nanocrystal characterization

**X-ray diffraction:** 1 mL of a stock solution of the nanoparticles in hexane was precipitated with addition of 2 mL of EtOH. The nanoparticle slurry was spotted onto a glass coverslip or silicon wafer multiple times until an opaque white film formed, and the sample was allowed to air dry completely. XRD patterns were obtained on a Bruker AXS D8 Discover GADDS X-ray diffractometer system with Co Kα radiation (λ= 1.78897 Å) from 2*θ* of 15 to 65°.

**Electron microscopy:** UCNPs were precipitated, resuspended in hexane to 10 nM, and 7 μL was dropped onto ultra-thin carbon film/holey carbon grid, 400 mesh copper (Ted Pella). Images of the nanoparticles were obtained using a Zeiss Gemini Ultra-55 analytical scanning electron microscope. Dark-field images were collected in transmission (STEM) mode with 30 kV beam energy. HR-TEM images were acquired on a JEOL 2100-F 200 kV field-emission analytical transmission electron microscope.

**Dynamic Light Scattering:** Aqueous nanocrystal size was determined by dynamic light scattering measurements on a Malvern Zetasizer. Samples were prepared from aqueous stocks by dilution with ddH_2_O to ~50 nM. Hydrodynamic diameters were determined by instrument software based on volume fittings.

**Optical characterization:** aUCNPs emission and lifetimes were characterized as functions of illumination intensity, illumination pulse duration, each with either 980 or 1550 nm excitation. Vials of hydrophobic aUNCPs dispersions in hexane (400 μL of the 0.68 μM) were placed above the imager array and excited with time-gated collimated lasers. The beam was positioned 2 mm above the surface of the imager.

INSITE samples were excited with a 980-nm wavelength-stabilized, single-mode, fiber-coupled laser diode (Qphotonics QFBGLD-980-500) followed by an adjustable collimator (Thorlabs ZC618FC-B) set to a beam diameter of 1.27 mm; or a 1550-nm single-mode, fiber-coupled laser diode (Qphotonics QFLD-1550-150S) collimated by an aspheric collimator (Thorlabs CFS2-1550-APC) with a beam diameter of approximately 0.3 mm. Both lasers were driven by a temperature-controlled mount driver (Arroyo Instruments 6310 ComboSource). Unless otherwise indicated, illumination parameters and laser settings used for INSITE for 980 nm and 1550 nm are given in Table S1.

Radiative lifetimes (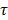) were modeled as a single exponential decay and were calculated by extracting decay profiles with a fixed moving integrating window (*T_int_*). Assuming the dark current intensity (*i*_d_) is constant over time, we derive the integrated pixel value *I*_A_(t) from the current density *i*(t):


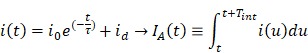



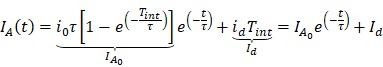


where τ and I_D_ are the emission decay lifetime and dark current level in the pixel, respectively 47. Dark current level was subtracted from waveforms.

**Effects of excitation (*T_exc_*) pulse duration on emission signal intensity**: To extract the excitation duration dependency, the aUCNPs were excited for increasing durations of time (*T*_exc_) and the emission intensity was measured. This duration represents how long the nanoparticles are illuminated with the excitation light source before the start of the time-resolved imaging sequence.

### Fabrication of PDMS micro-wells

We fabricated 500-micron cubic wells for aUCNP coating purposes by micro-patterning wells into a PDMS substrate. We subsequently coated the internal surface of the PDMS micro-well with 10 μL of 0.68 μM 16-nm NaEr_0.6_Yb_0.4_F_4_ core/shell aUCNPs in hexane, giving a final surface concentration of 5 pM/mm^2^ of adsorbed UCNP. This aUCNP-coated structure was imaged with INSITE using a 1550 nm laser at 60 W/cm^2^.

### Imaging aUCNPs in tissue with INSITE

Animal experiments were conducted according to protocols approved by the UCSF Animal Care and Use Committee. Tumor-bearing mice were generated by subcutaneous implantation of prostate cancer (LNCaP) cells bilaterally over the flank of a nude male mouse (5-week-old, Tarconic Farms). Tumors were allowed to grow to 1 cm in diameter. Mice were anesthetized and single tumors were injected with injected with 25 μL of 250 nM polymer-encapsulated 26-nm (16-nm cores with 5-nm shells) NaEr_0.8_Yb_0.2_F_4_ core/shell aUCNP dispersions on the left side tumor of the mouse (ventral left), and the right side tumor was used as a reference and negative control. Mice were imaged with an IVIS Spectrum (In Vivo Imaging System, PerkinElmer) equipped with a 980-nm continuous wave laser (Qphotonics) and 780-nm short pass filter (Chroma), to reject 980-nm interference on the IVIS camera. Emission was collected from 650-670 nm in the ^4^F_9/2_ Er^3+^ band using 2.5-s integration times.

Tumors with aUCNPs were excised and imaged by INSITE with a 1550-nm pulsed laser (Qphotonics) scanned across the sample in 300-μm increments. Excised tumors were also imaged under a widefield microscope (Leica DMIRB) customized to image aUCNPs using a 980 nm laser (Qphotonics) at 1 W/cm^2^.

## Results and Discussion

### Optimization of UNCPs for INSITE Imaging

**Figure 1:**
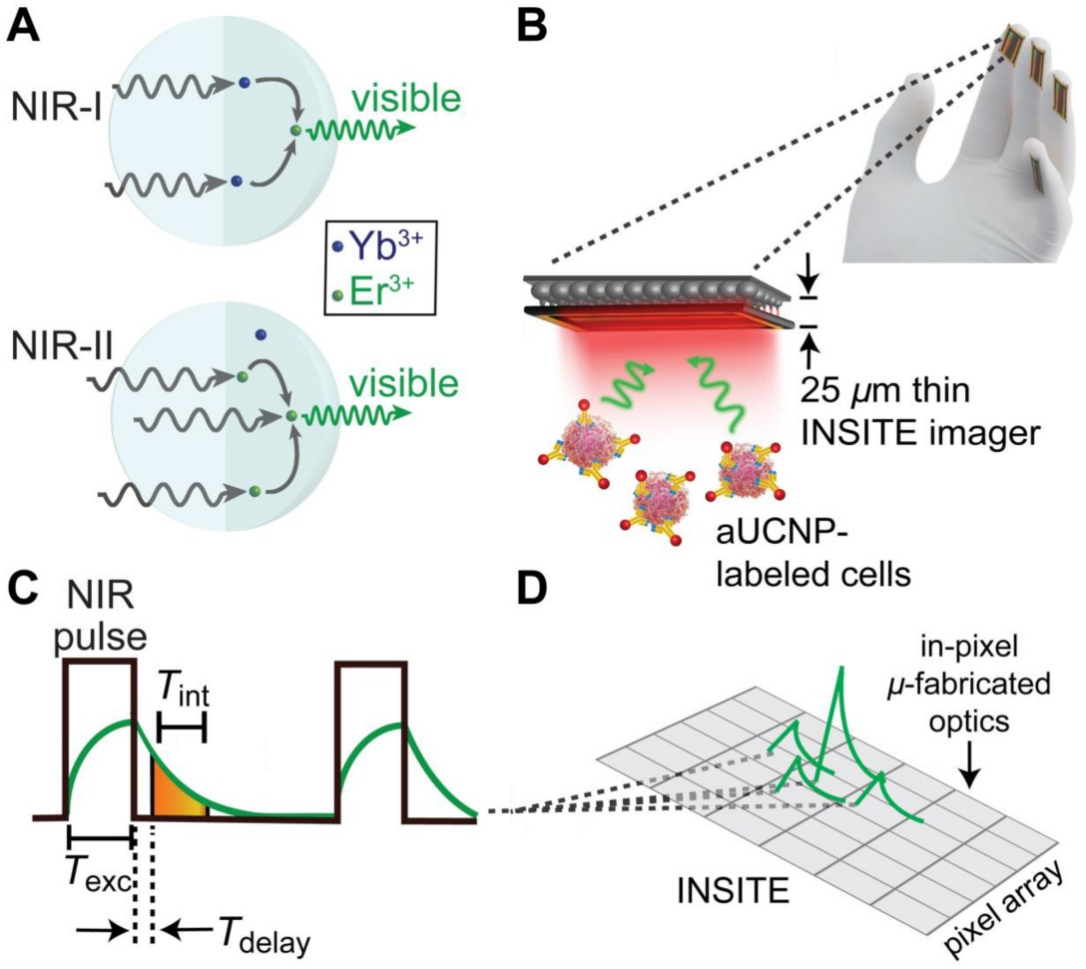
** INSITE imaging of upconverting nanoparticles. (A)** Multiphoton energy absorption, transfer, and emission in Yb^3+^/Er^3+^-based aUCNPs following either NIR-I (980 nm) or NIR-II (1550 nm) excitation**. (B)** Cartoon of INSITE directly integrated onto surfaces, such surgical glove. **(C)** Diagram of the time-resolved image acquisition scheme. *T*_exc_, pulse excitation time; *T*_int_, emission signal integration time; *T*_delay_, time gating delay. (D) Angle-selective gratings used to achieve lensless image acquisition and block obliquely incident background light.

**Figure 2:**
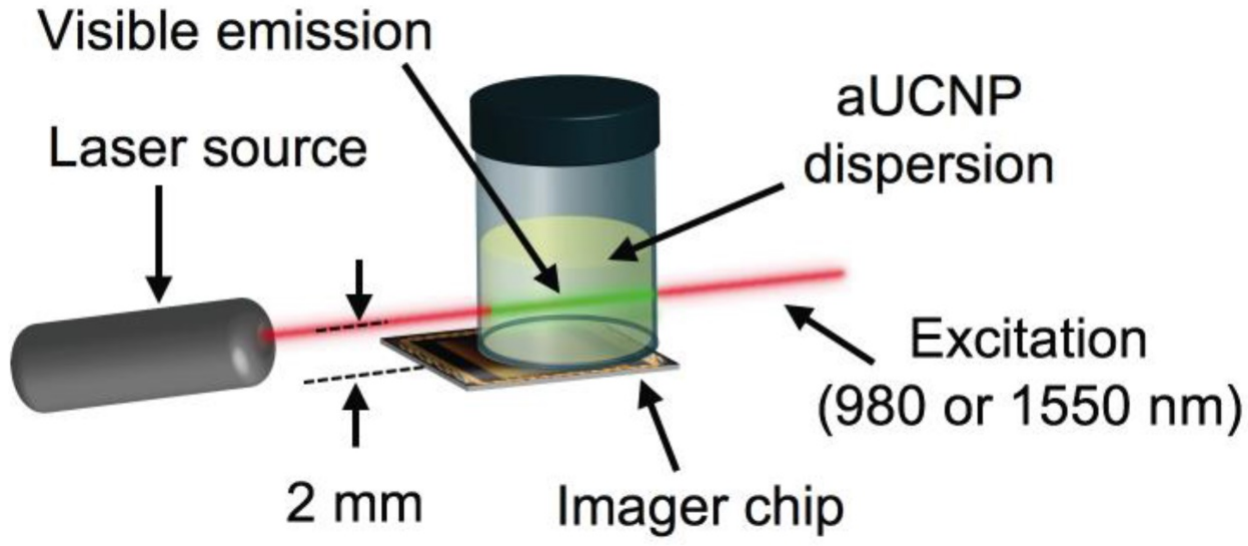
INSITE configuration for imaging of aUCNP dispersions. Laser beam width is 300 μm.

**Figure 3:**
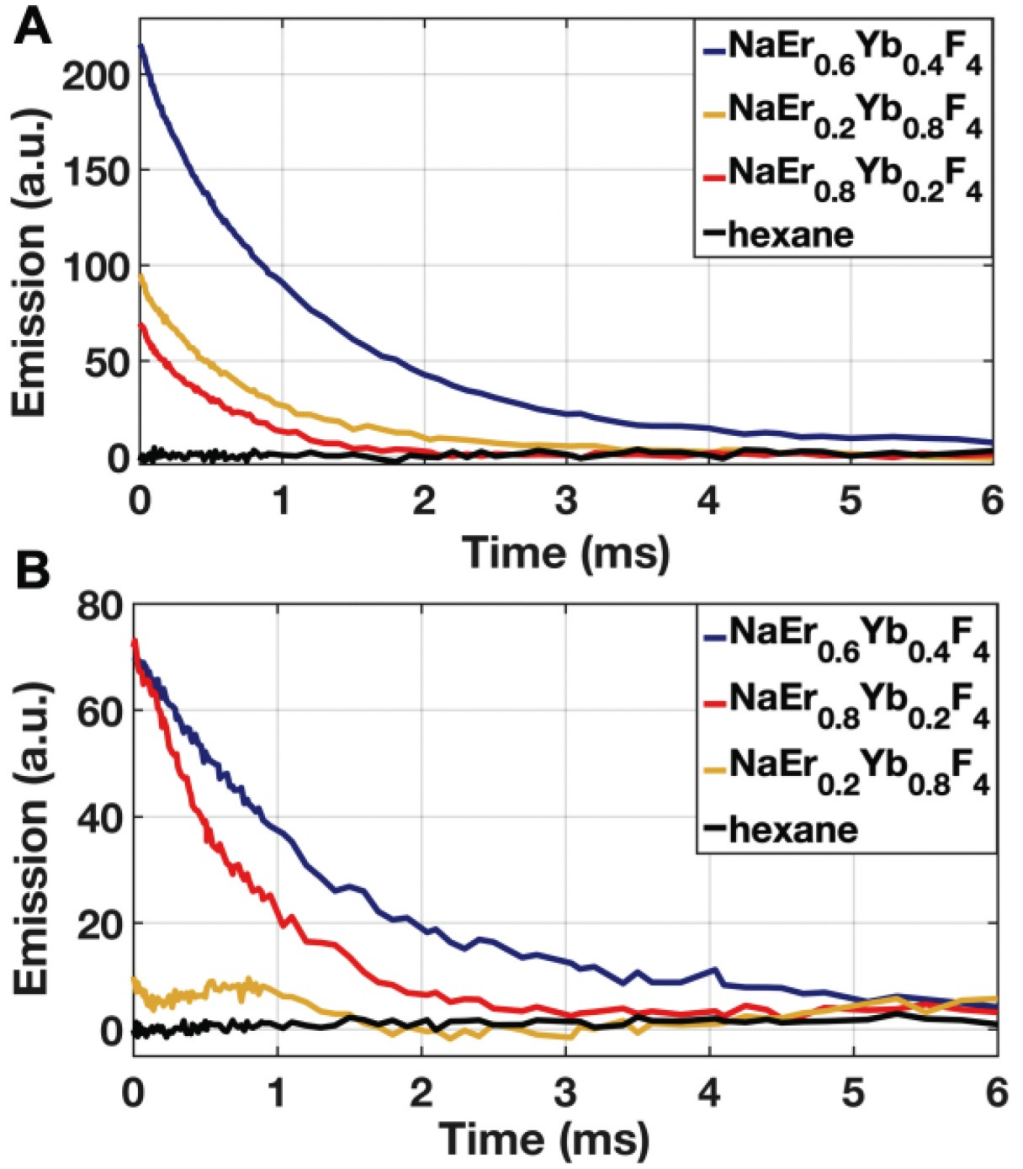
Emission decays of aUCNPs (*T_i_*_nt_ = 1 ms, *T*_exc_ = 5 ms) as measured by pixel output at (A) 8 W/cm^2^ of 980 nm excitation, or (B) 60 W/cm^2^ of 1550 nm excitation. *T*_int_ is integation time; *T*_exc_ is duration of exciation light pulse.

**Figure 4:**
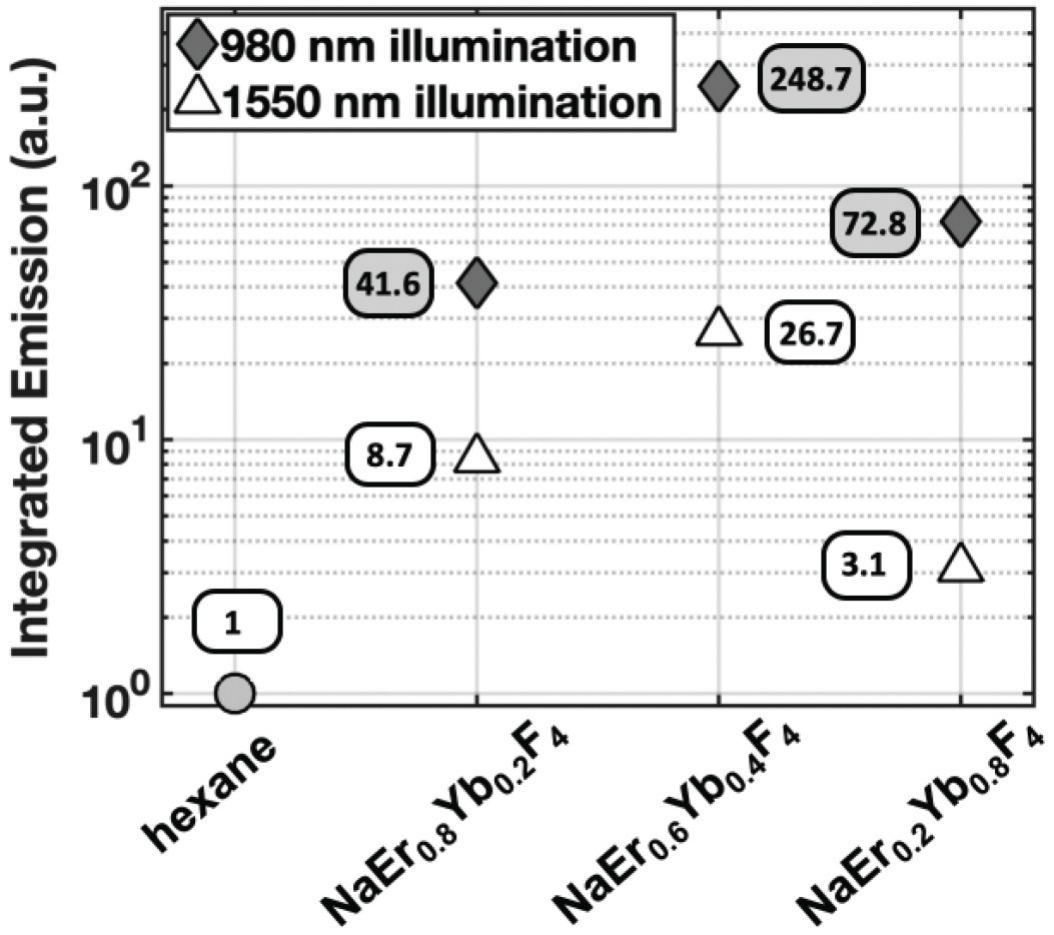
Integrated visible emission as a function of aUCNP composition at either 980 and 1550 nm excitation, both with 8 W/cm^2^ excitation power density. Hexane blank is without aUCNPs.

To determine the optimal composition of aUCNPs for use with INSITE (Figure 1B), we synthesized a series of core/shell aUCNPs with varying Yb^3+^ and Er^3+^ content to measure emission using either 980 nm or 1550 nm photoexcitation. aUCNP cores (8 nm) were synthesized with 20/80, 40/60, or 80/20 Yb^3+^/Er^3+^ ratios and overgrown with inert 4-nm shells 37. These hydrophobic nanocrystals were characterized by transmission electron microscopy (TEM) to measure size, dispersity, and crystallinity (Figures S1-2). With these nanocrystals in the experimental setup (Figure 2), we measured the initial emission intensity, 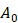, luminescence lifetime, 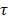, (Figure 3) and total integrated emission count, 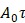, at a given power density (8 W/cm^2^ at either 980 and 1550 nm) (Figure 4). Measured decays for varying aUCNP compositions range from 600 μs to 1.3 ms at either 980 nm or 1550 nm excitation. These values are longer than measured 37 or calculated, but consistent with UCNP power-dependence given the lower excitation powers used here.

While most Yb^3+^/Er^3+^ upconversion is nominally a 2-photon process with following 980-nm excitation of the Yb^3+ 2^F_5/2_ manifold, 1550 nm excitation of the Er^3+ 4^I_13/2_ manifold leads to upconversion via a nominal 3-photon process (Figure 1a). For all aUCNP compositions, NIR-I excitation produces a stronger signal than with NIR-II, consistent with significantly higher efficiency of 2-photon versus 3-photon upconversion processes 48. At both excitation wavelengths, NaEr_0.6_Yb_0.4_F_4_ aUCNPs are the brightest composition (Figure 4) as measured by INSITE, although single nanoparticle studies at low 980-nm fluences have shown little difference in absolute brightness between aUCNP compositions 37. Differences in brightness here may be due to subtle variations in radiative lifetime, since the INSITE 100-μs time gating (*T*_delay_) will cause greater losses in aUCNPs with faster upconversion. Kinetic modeling has shown that Yb^3+^/Er^3+^ lifetimes are complex and deeply power-dependent 42, 48 but alloyed compositions do show kinetic differences in both green (^4^S_3/2_, ^2^H_11/2_) and red (^4^F_9/2_) Er^3+^ emission 48.

Long decay lifetimes enable time-resolved imaging in modern CMOS technologies, and alleviate the need for optical filters entirely. While time resolved imaging has been demonstrated with organic and protein fluorophores, their nanosecond radiative lifetimes 17 make large, dense array based imaging impossible, as arrayed CMOS sensors cannot readily detect on timescales shorter than tens of microseconds 49, 50. Although single photon avalanche diodes that require specialized fabrication processes have been demonstrated to operate at these timescales 51, we require a massively parallel array-based approach for larger spatial coverage, high fill factor, and adequate spatial resolution necessary for efficient chip-based imaging. Consequently, a chip-based imager using time resolved imaging requires optical probes with microsecond lifetimes, such as upconverting nanoparticles 37, 47, 48. The design of UCNPs with a core-shell prevents rapid quenching, as tested here, significantly elongating the decay lifetime. The 0.6-1.3 ms decay lifetimes open the door to time-resolved imaging in an array-based CMOS imager.

The chip-based time-resolved imaging method takes advantage of these uniquely long emission lifetimes the aUCNPs studied here, and alleviates the need for high performance frequency-selective (color) filters by separating the emission and excitation signals in the time domain rather than in frequency domain, a strategy that can be implemented in modern high-speed integrated circuit design. In a chip-based imager, we implement this by briefly pulsing the excitation light (*T_exc_* = 5 ms duration) while the imaging pixels are not integrating. After the excitation light is turned off, the pixels are turned on, and integrate the emission signal from the aUCNPs for 1 ms (Figure 1C). Since there is no background excitation light at this time, the need for an optical filter is eliminated. The illumination and signal acquisition scheme (Figure 1C), where the excitation light source is pulsed for *T_exc_*, and subsequently turned off, after which the emission signal from the aUCNPs is acquired and integrated by the imager. Any interference and unwanted signal caused by the excitation light can be rejected by delaying (*T*_delay_) the integration window start point.

### aUCNP brightness as a function of NIR-I and NIR-II excitation intensity and pulse duration

In order to minimize overall imaging time for rapid imaging which consists of illumination, integration and chip readout, and duration of optical exposure for tissue, we investigated the optimal time for the illumination pulse. We find increases in emission intensity for all three aUCNP compositions with increasing *T*_exc_ up to a threshold of 5 ms, after which emission intensity plateaus, and longer integration pulses cause a measurable increases in detector noise (Figure S3). Pulsed illumination is safer than continuous wave (CW) illumination, and human maximum permitted exposures are ~3 orders of magnitude higher for pulsed versus CW at both NIR-I and NIR-II wavelengths 43. These 5-ms pulses then permit significantly higher excitation fluences, with resulting increased sensitivity and signal-to-background ratios.

To quantify the relationship between excitation light intensity and emission intensity, all 3 aUCNP compositions were excited at increasing illumination intensities at both 980 nm and 1550 nm, and emission intensity was measured by INSITE (Figure S4). UCNP excitation is nominally a 2- and 3-photon process, but the relationship between illumination power density and emission intensity is complex and varies with both excitation and detection schemes 48. While varying illumination intensity over orders of magnitude below saturation elicits non-linear responses 37, 39, the linear response we observed here may be due to the small range of illumination intensities, time-gating, and pulse sequence.

Optical probes excited in the NIR-I and NIR-II spectrum are ideal for both *in vivo* imaging and CMOS-based tissue imaging chips. NIR-I and NIR-II illumination enables deeper tissue penetration and lower scatter than illumination in the visible spectrum, common to most optical labels. Furthermore, aUCNP excitation in the NIR-I and NIR-II regions is particularly attractive for CMOS-based imagers, as it minimally interacts with silicon. At these wavelengths, the thin imager itself is effectively transparent and minimal background and noise is generated in the imager by the illumination light.

Despite the stronger aUCNP emission signal with NIR-I excitation, NIR-II light is more weakly absorbed by silicon and may be advantageous for chip-based microscopes. The silicon bandgap energy corresponds to ~1100 nm 52 and at wavelengths above that, photons pass through with relatively little interaction. At shorter wavelengths, particularly in the visible, photon interaction with the chip can introduce substantial background signal. Excitation with 980 nm light (just below the silicon bandgap) produces a small background signal with INSITE (Figure 4 and Figure S4, hexane), while for 1550 nm illumination (substantially above the silicon bandgap), such background is absent. Because background substantially affects signal to noise ratios, using higher excitation powers at 1550 nm to compensate for lower aUCNP emissions may ultimately lead to better image quality.

Upconversion, along with NIR illumination and long decay times, enables high contrast and sensitivity imaging by eliminating autofluorescence background. Upconversion places the emission photos in the high responsivity (sensitivity) range of silicon-based detectors, enabling imaging of these nanoparticles 37. In comparison, light from a conventional fluorophore excited in the NIR I or NIR II spectrum, will emit a lower energy (longer wavelength) photon, which would pass through silicon undetected.

### INSITE imager design

In order to determine whether an ASIC can image optical labels without the use of conventional focusing lenses and optical filters, we designed and fabricated an imaging array capable of time resolved imaging (Figure 1)**.** Absent optics, the imaging chip can be easily thinned to just 25 microns and placed directly on tissue, increasing sensitivity through proximity - capturing light from optical labels before it diverges. The key to this platform is the transformation of molecular imaging from the color (frequency) domain to the time domain enabled by aUCNPs 37. We accomplish this using synergistic design of modern integrated-circuits and upconverting nanoparticles. A key advantage of using CMOS-based imaging platform is the ability to integrate in-pixel electronics enabling signal processing directly on-chip, eliminating the need for optical lenses. This imager addresses both elimination of lenses and filters simultaneously, making it possible to obtain optical images with a much smaller form-factor.

### INSITE spatial resolution

**Figure 5:**
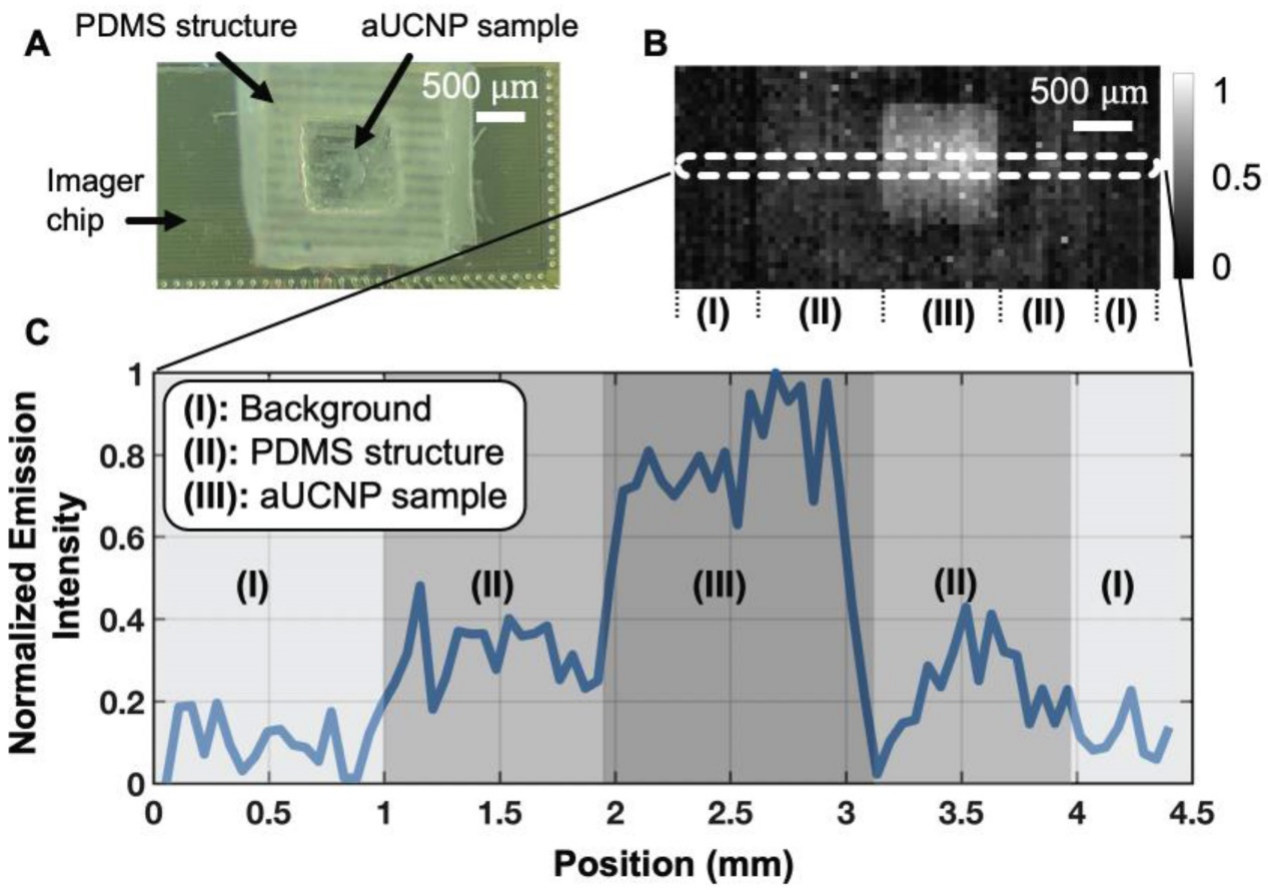
(A) Photograph of CMOS contact imager and PDMS micro-well holding aUCNPs, fabricated for this study. (B) Time-resolved image of the aUCNP-coated surface of the micro-well. Normalized emission intensity shown as in grayscale legend. Section (I) is background, (II) is outer PDMS with aUNCPs diffused into PDMS, and (III) is microwell with aUCNP sample. (C) Cross-section emission profile for the three regions in (B).

To determine the imaging quality achievable with a 25-micron thin microscope, we used a 1550 nm excitation source to excite the aUCNP-coated microstructure and acquired the image using time-resolved imaging. The custom-fabricated PDMS micro-well and the CMOS imager demonstrate that INSITE is able to resolve the spatial features of the micro-well (Figure 5A and 5B) with nearly single-pixel sharpness (55 μm), translating into a spatial resolution performance sufficient for detecting microscopic residual disease. A cross-section of the acquired signals (Figure 5C), shows three different regions of the image. Aside from the micro-well and the background, the intermediate zone represents the PDMS surrounding the micro-well. Due to the porosity of the PDMS, small amounts of aUCNPs diffuse into the surrounding area, generating a small signal in this area. For a concentration of 10^12^ aUCNPs per mm^2^, the measured average signal-to-background ratio is 6.5. The background is dominated by the dark pixel current, which can ultimately be subtracted out.

INSITE achieves this spatial resolution without the use of conventional lenses through both proximity to the tissue sample and direct integration of on-chip microfabricated collimators, and is limited only by the pixel size. INSITE uses angle-selective gratings (ASG) to improve spatial resolution with chip-based imaging 22, 45. ASG are arrays of microcollimators fabricated directly on each pixel using only the inherent metal interconnect layers common to all CMOS process - obviating the need for any postprocessing and not adding any thickness to the imager itself. The versatility of CMOS fabrication technology has led to the on-chip integration of a variety of optical components such as wavelength-selective optical filters 21, 53 that could be tuned to be compatible with quantum dot applications 54, or stacked diffraction gratings for lensless 3D imaging 55 to reject angled incoming light and decrease blur in the image. Other lensless imaging platforms have also been reported in 24, 26, 56 that leverage computational techniques. As demonstrated here, the elimination of optical filters and focusing optics enables placement of the custom designed INSITE imaging chip directly against the sample itself, capturing light before it diverges, achieving both spatial resolution and increased sensitivity without optics.

### INSITE tumor imaging

**Figure 6:**
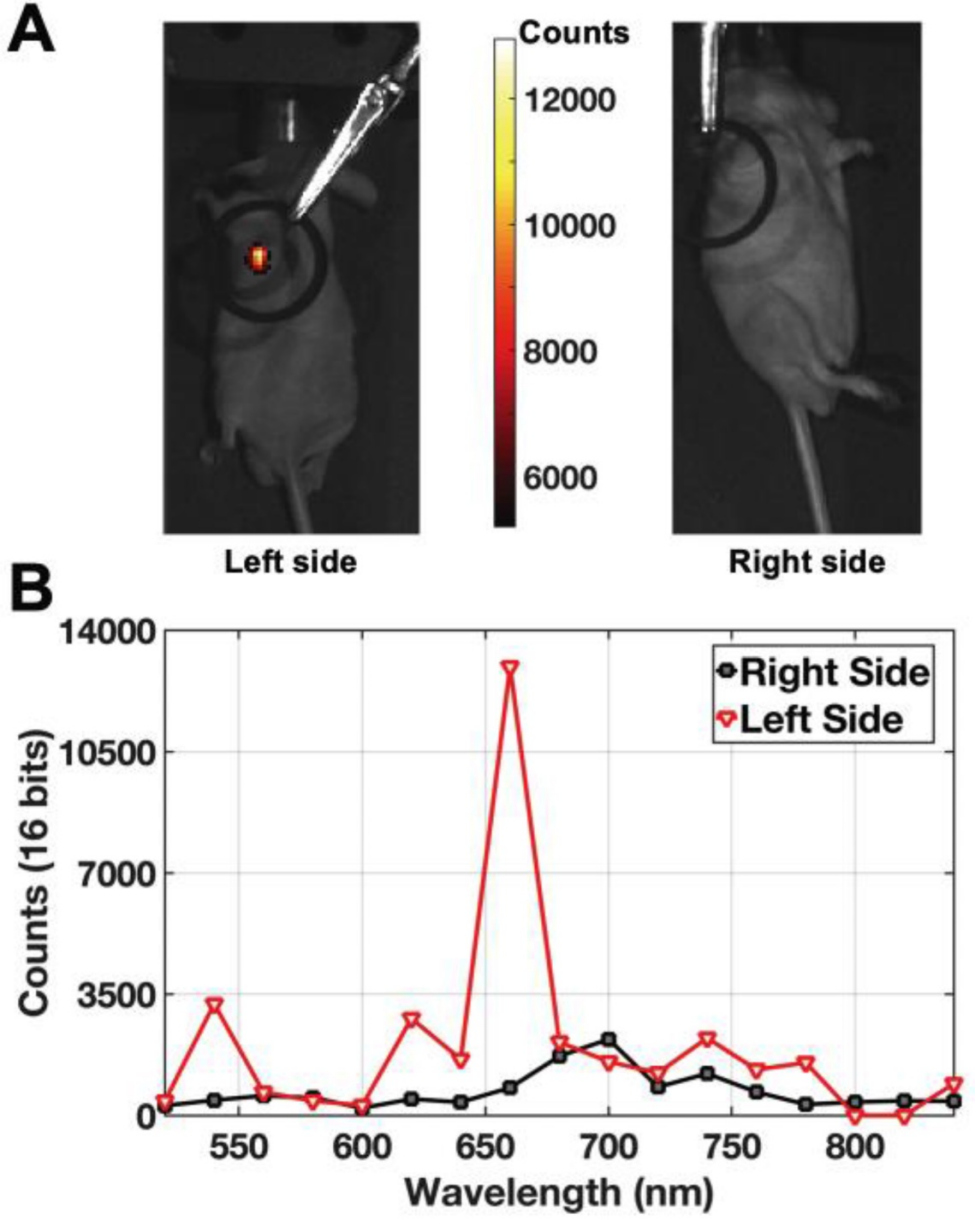
Live mouse images of intratumorally-injected NaEr_0.8_Yb_0.2_F_4_ aUCNPs with 8 W/cm*^2^* 980 nm excitation. (A) Images of aUCNPs-injected into mouse prostate tumor (*left*) and non-injected side (*right*). Emission intensity as in colored legend. (B) Measured emission spectrum of the injected and non-injected sides, showing tumor-specific Er^3+^ emission bands at 545 and 655 nm.

To determine the applicability of INSITE to tissue imaging, we injected a prostate tumor with aqueous 250 nM polymer-encapsulated 26-nm NaEr_0.8_Yb_0.2_F_4_ aUNCPs. Hydrophobic, as-synthesized nanocrystals were transferred to water by encapsulation within amphiphilic polymers terminated with short PEG chains 57, 58. Previous work with these and similar amphiphilic polymers 39, 46 has shown that nanoparticle brightness is fully preserved by retention of the oleic acid surfactant layer. Polymer encapsulation increases nanoparticle diameters by 3-5 nm (Figure S2 and 59) and present multiple surface carboxylates that, along with the PEG chains, may minimize non-specific endocytosis 57, 58. aUCNP-injected mice were imaged a custom-modified IVIS imager using NIR-I illumination, showing colocalization of the tumor and aUCNPs (Figure 6A). Images of a tumor on the contralateral side of the mouse without aUCNP injection shows no measurable visible emission (background dark current only). To ensure aUCNPs were being imaged, the spectrum of the acquired emission signal was measured (Figure 6B), displaying the characteristic emission spectrum of the nanoparticles, with the two major visible emission bands of the aUCNPs at 545 nm and 655 nm. Using the aUCNP emission band at 650-670 nm, the signal-to-background ratio is 15.

**Figure 7:**
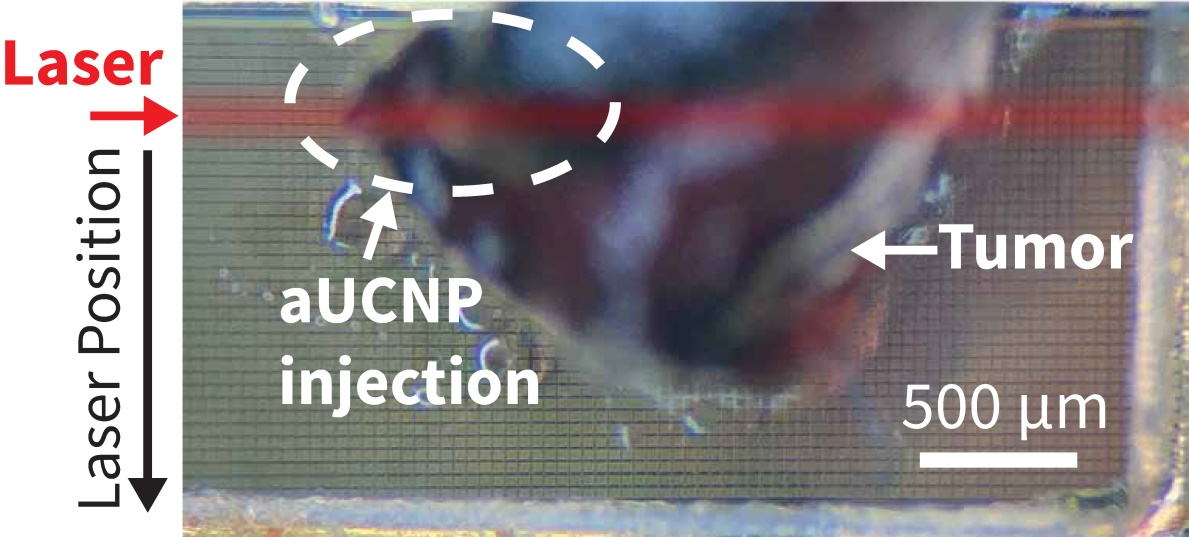
Photograph of the tumor injected with aUCNPs (left side of mouse, from Figure 6) is excised and placed directly on INSITE for imaging. The area within the excised tissue where the aUCNPs are located (aUCNP spot) is circled. The remainder of the tissue does not contain aUCNPs. The path of the illumination laser is drawn in red. *This is for illustrative purposes only, and is not an image of the actual laser beam, which is not visible.

**Figure 8:**
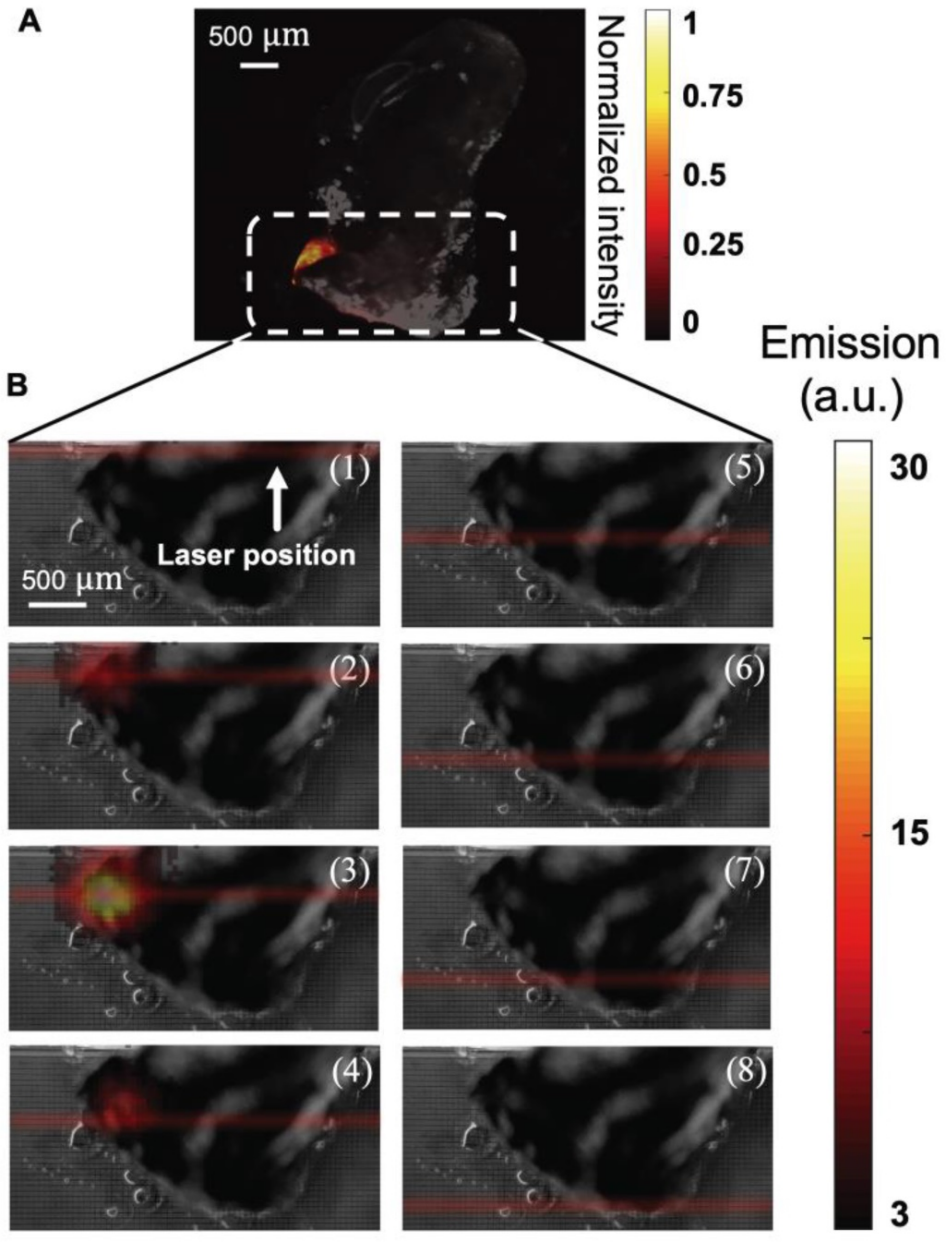
(A) Microscope image of excised tumor injected with 25 μL of a 250 nM aqueous dispersion of aUCNPs, excited with 1 W/cm^2^ 980 nm, showing distinct localization of aUCNPs. (B) Images of NIR-II laser scanning of tumor, from top to bottom in increments of 300 μm (numbered 1-8). At each position, an image of the tumor sample is acquired with INSITE using a 5 millisecond-pulsed 60 W/cm^2^ 1550-nm laser for illumination.

To determine if the aUCNPs injected into the tumor can be visualized with INSITE alone, we next excised the injected tumor and imaged with the INSITE chip imager. Figure 7 shows the photograph of the excised tumor sample on the 25-micron imaging chip. For reference, we image the excised tumor on a microscope (Figure 8A), and the image acquired with INSITE reveals a distinct area of aUCNPs in the excised tissue (Figure 8B) with a signal to background ratio of 9, closely matching the performance of the IVIS imager, which incorporates high performance optical filters and a cooled CCD camera. Results from these experiments demonstrate that the ultra-thin time-resolved CMOS imager, custom designed to image engineered aUCNPs, is capable of imaging within tissue, with no background autofluorescence and with little to no additional interference from the excitation light. More extensive work in toxicology and biodistribution with immunotargeted aUCNPs will be needed to assess the suitability for these nanoparticles for tumor targeting in mice or human tissue.

We have designed a 25-micron thin microscope, INSITE, and optimized it for imaging aUCNPs with NIR-I or NIR-II excitation. UCNPs have been successfully used for *in vivo* animal imaging 60-64, but to date the imagers remain too bulky and illumination power requirements are too high to be used in minimally invasive surgeries. Using complementary design of imager and optical label, INSITE synergistically integrates the advantages of both modern integrated circuit design with nanoparticle engineering. Leveraging the engineered long luminescent lifetimes and upconversion of aUCNPs we demonstrate the optimal aUCNP composition for chip-based time-resolved imaging. We custom design our chip-based imager around the excitation and emission wavelengths of aUCNPs and their decay time constants. This eliminates optical filters and the requisite focusing optics without sacrificing background rejection. Pulsed illumination allows a 200x-1000x increase in instantaneous optical illumination power, while still respecting ANSI safety limits. Absent optics, the INSITE platform is thinned to just 25 microns at which point it becomes flexible, allowing integration on virtually any planar or curved surgical instrument without disruption of form-factor. On-chip integration of both optical sensing and signal processing enables conversion of an optical signal to an electronic one (and amplification) at the point of imaging - enabling only thin flexible wires to transmit power and data from the chip, achieving a level of flexibly and maneuverability not achievable with current approaches. The scalable design, inherent to array-based integrated circuits allows further customization to any device or tumor bed size, while the parallel operation of the imaging array ensures imaging time remains constant, regardless of size. This maintains the rapid imaging needed for seamless intraoperative evaluation of large surface areas. Future form-factors for *in vivo* imaging may fully integrate illumination with optical detection, such as by surrounding the imaging chip with laser diodes, or leveraging the transparency of silicon above 1100 nm to illuminate tissue via laser diodes affixed to the chip back-side. Conjugation of aUCNPs to molecules targeted to tumor cells can further enhance tumor specificity of these imagers, and INSITE has the potential to significantly reduce the rate of positive margins in cancer surgeries, significantly impacting patient outcomes across the cancer spectrum.

## Conclusion

This work provides a new strategy to bring NIR-I and NIR-II excitation-based imaging, with high sensitivity, into intraoperative imaging by introducing a time-resolved contact CMOS imaging array that no longer requires optics to resolve the image and can easily achieve a surgically-compatible form-factor. The integration of probe design, in terms of long-lifetime aUCNPs, with microscope engineering represents an initial foray into co-design of nanoparticles and imagers, and can be further optimized through iterative rounds of optimization for each element. The scalability inherent to CMOS technology will allow fabrication of larger arrayed image sensor to achieve greater spatial coverage, while preserving spatial resolution and imaging speed, than current imaging platforms.

## Abbreviation

INSITE: immunotargeted nanoparticle single-chip imaging technology
UV: ultraviolet
UCNP: upconverting nanoparticle
aUCNP: alloyed upconverting nanoparticle
PDMS: polydimethylsiloxane
IR: infrared
NIR: near infrared
CMOS: complementary metal-oxide-semiconductor
LNCaP: lymph node carcinoma of the prostate
CW: continuous wave
OA: oleic acid
MOM: metal-oxide-metal
ANSI: American national standards institute
MRD: microscopic residual disease
ASIC: application-specific integrated circuit
ODE: 1-octadecene
ASG: angle-selective gratings
