# Supplementary material for: A 25 micron-thin microscope for imaging upconverting nanoparticles with NIR-I and NIR-II illumination
Source: Theranostics. 2019 Oct 18;9(26):8239–52. doi: 10.7150/thno.37672 (PMC6857055; doi:10.7150/thno.37672)
Supplement: Supplementary file 1 — Supplementary figures and tables. [file thnov09p8239s1.pdf]

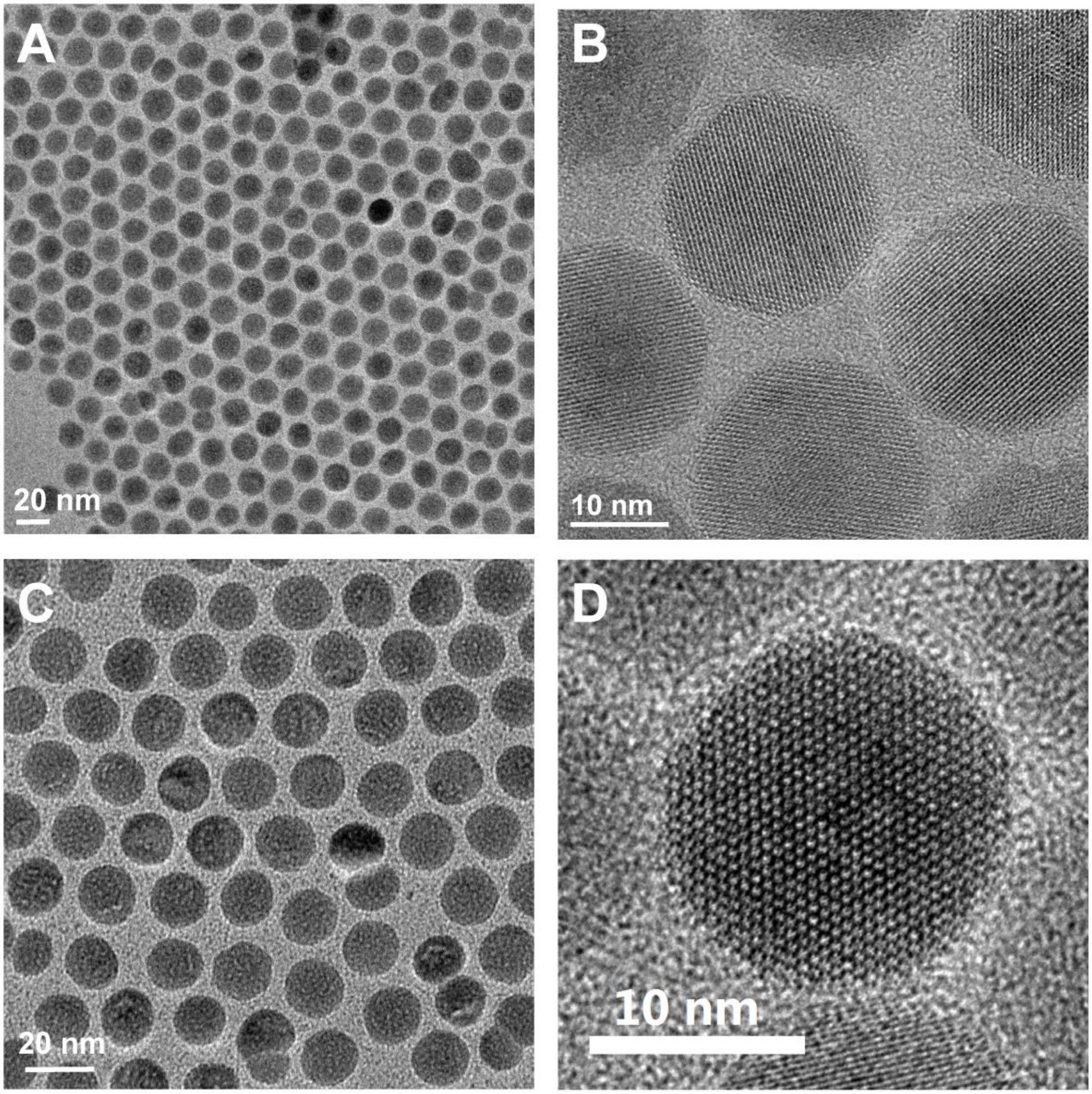

Figure S1. Characterization of aUCNP size and morphology by transmission electron microscopy (TEM).
(A) TEM and (B) high resolution TEM of hydrophobic 8-nm ß-phase $NaEr_{0.6}Yb_{0.4}F_4$ aUCNPs with 4-nm $NaYF_4$ shells. (C) TEM and (D) HR-TEM of hydrophobic 8-nm ß-phase $NaEr_{0.2}Yb_{0.8}F_4$ aUCNPs with 4-nm $NaYF_4$ shells.

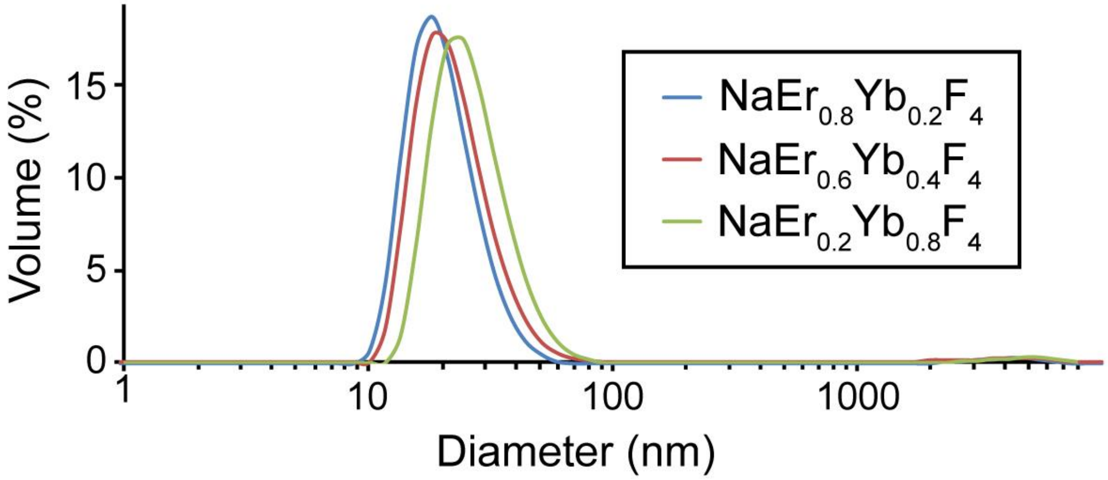

Figure S2. Sizes of aqueous aUCNPs determined by dynamic light scattering (DLS). Hydrodynamic diameters of 16-nm core/shell aUCNPs encapsulated within PMAO amphiphilic polymers.

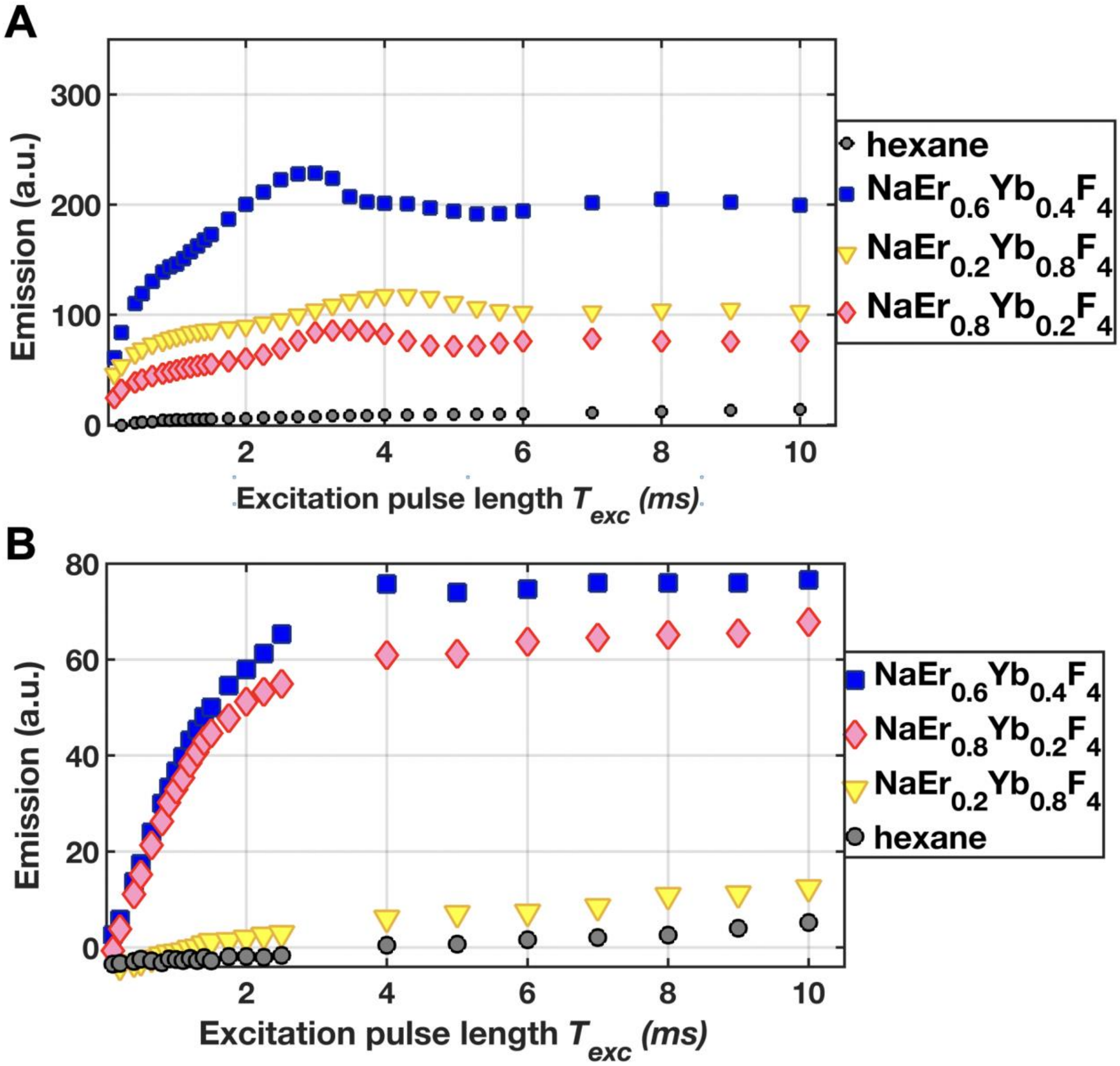

Figure S3. Measured emission intensity of aUCNPs at different excitation durations (T_{int} = 1 ms).

(A) NIR-I excitation process with 8 W/cm^2 980 nm excitation.

(B) NIR-II excitation process with 60 W/cm^2 1550 nm excitation. T_{exc} is the excitation illumination pulse duration.

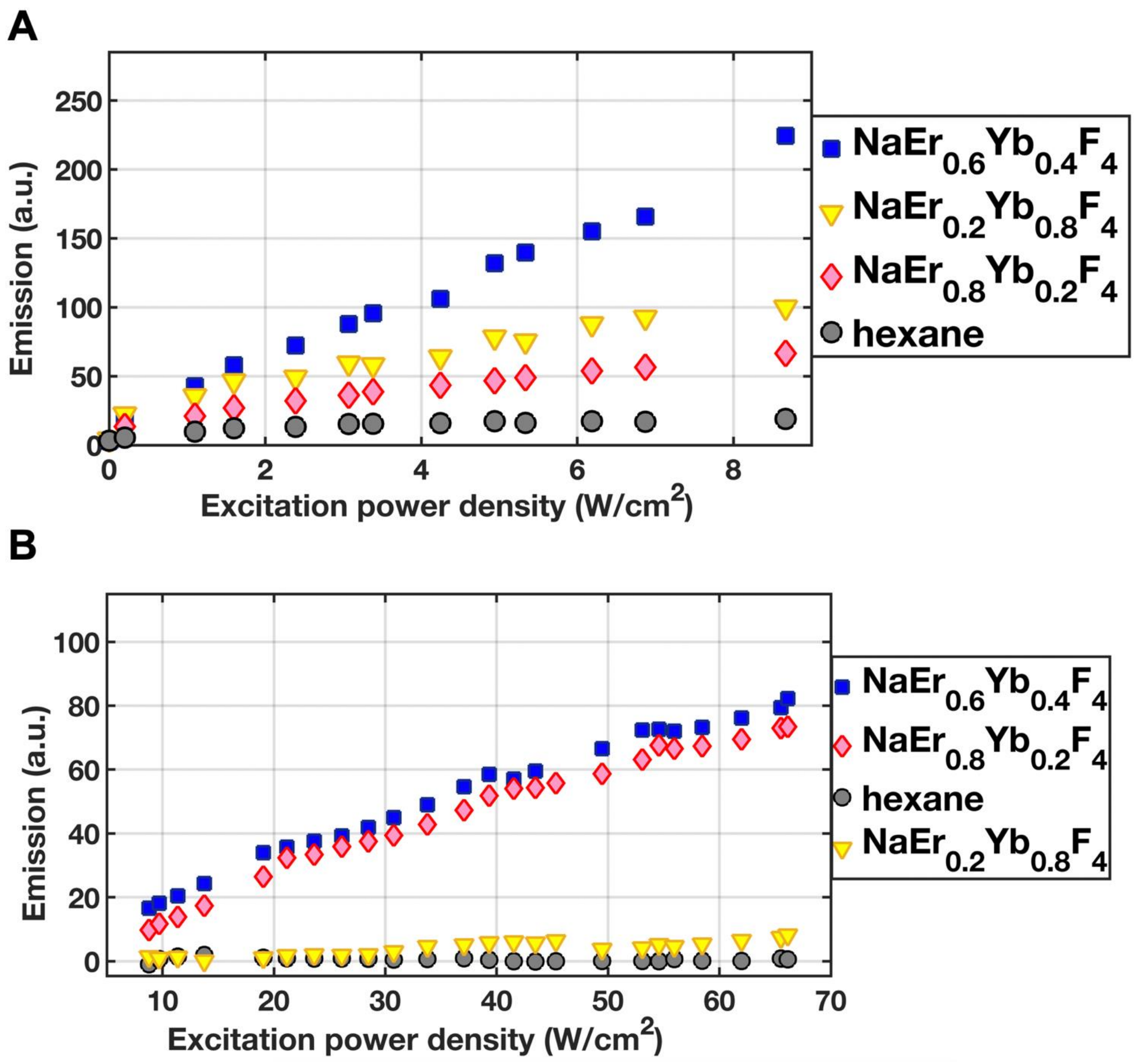

Figure S4. Measured emission intensity of aUCNPs at different power levels (T_{int} = 1 ms, T_{exc} = 5 ms).

(A) NIR-I excitation process with 980 nm excitation.

(B) NIR-II excitation process with 1550 nm excitation.

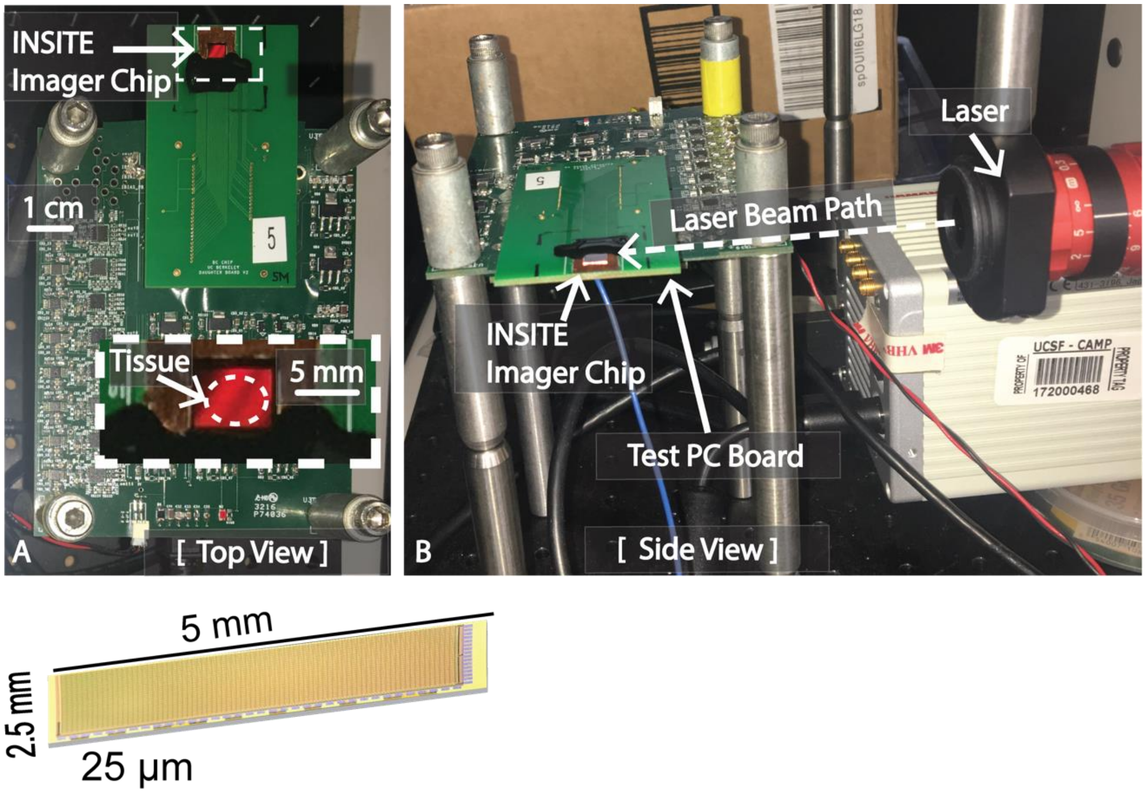

C INSITE Imager chip

Figure S5. INSITE Test setup.
(A) Top view of INSITE imaging chip on test board. (Inset: Zoom of INSITE imaging chip. The tissue sample is placed directly on the imaging chip.)

(B). Side view of INSITE test setup, with laser light illuminating tissue sample. Functions performed by the PC Board can be decoupled from the imaging chip through connecting wires.

(C) Zoom of INSITE Imaging chip. Thickness of chip after thinning is 25 microns.

Wave-length (nm)	Average Power density (W/cm^2)	Pulse duration (ms)	Pulse repetition rate (Hz)	Peak Intensity (W/cm^2)[b]	Total pulse energy (J/cm^2)	MPE[a] (J/cm^2)	Maximum Power density for 5-ms pulse (W/cm^2)[a]	Focal spot area (mm^2)
**980**	5.2	5	130	8	0.04	1.06	212	0.3
**1550**	39	5	130	60	0.3	1	200	0.3

[a]MPE, Maximum Permitted Exposure for human tissue, calculated as described in ((American National Standards Institute 2014))
[b]pulse energy/(pulse duration * focal spot area)

Table S1. Illumination parameters and safety limits

Bibliography

American National Standards Institute 2014. *American national standard for the safe use of lasers, ANSI Z136.1-2014*. New York: National Standards Institute.
